# A designed peptide disrupting viral protease cleavage restores cGAS-DNA phase separation and type I interferon responses

**DOI:** 10.1371/journal.ppat.1014291

**Published:** 2026-06-01

**Authors:** Hongyan Yin, Zhenchao Zhao, Haiwei Wang, Xin Li

**Affiliations:** 1 State Key Laboratory of Veterinary Public Health and Safety, College of Veterinary Medicine, China Agricultural University, Beijing, China; 2 State Key Laboratory of Animal Disease Control, Harbin Veterinary Research Institute, Chinese Academy of Agricultural Sciences, Harbin, China; 3 Department of Respiratory Medicine, Center of Infectious Diseases and Pathogen Biology, Key Laboratory of Organ Regeneration and Transplantation of the Ministry of Education, State Key Laboratory for Diagnosis and Treatment of Severe Zoonotic Infectious Diseases, The First Hospital of Jilin University, Changchun, China; Washington State University, UNITED STATES OF AMERICA

## Abstract

Seneca Valley Virus (SVV) 3C protease is essential for viral polyprotein processing and virion assembly. Meanwhile, it has evolved to cleave and antagonize the multiple innate immune proteins, enabling viral immune evasion. Inhibitors of 3C protease are therefore powerful antiviral agents. Among these, antiviral peptide inhibitors hold particular promise because of their high specificity, strong efficacy, and broad-spectrum activity, and minimal side effects. Here, we developed a dimerization-dependent red fluorescent protein (ddRFP) biosensor system to screen anti-SVV 3C peptides and identified a substrate-competitive decapeptide (P5) that markedly suppresses 3C protease activity. P5 inhibited 3C-mediated cleavage of multiple key immune proteins, including porcine cGAS (pcGAS), porcine Gasdermin A (pGSDMA) and porcine Pro-IL-1β (sPro-IL-1β). Mechanistically, P5 directly interacted with the catalytic His48 site of 3C protease through hydrogen bonding. Remarkably, P5 restored the formation of cGAS-DNA liquid-liquid phase separation (LLPS) by competitively blocking 3C cleavage activity, thereby enhancing cGAS activity and downstream antiviral interferon signaling. Furthermore, P5 demonstrated favorable cellular permeability, low cytotoxicity, good stability and robust antiviral activity. Our findings establish P5 as a highly promising peptide inhibitor of SVV 3C protease with strong translational potential.

## Author summary

Seneca Valley virus (SVV) 3C protease, essential for both viral replication and cleavage of host immune proteins, represents a high-value target for antiviral development. We identify P5, a substrate-competitive decapeptide that directly inhibits 3C protease by binding to the catalytic His48 residue within its active site. This interaction effectively blocks 3C-mediated cleavage of multiple key host immune proteins, thereby conferring antiviral activity. Importantly, P5 restores cGAS-dependent liquid-liquid phase separation (LLPS) by preventing 3C-cGAS interaction, reactivating downstream interferon signaling. This dual-action peptide exhibits favorable drug-like properties, including cellular permeability for intracellular targeting, low cytotoxicity, and robust antiviral efficacy. Collectively, P5 represents a promising multi-functional therapeutic lead for the treatment of SVV infection.

## Introduction

Seneca Valley Virus (SVV) is an emerging pathogen associated with porcine idiopathic vesicular disease (PIVD), underscoring the urgent need for effective therapeutic strategies [1–3]. The SVV 3C protease plays an essential role in both viral replication and immune evasion, making it an attractive target for drug development [4,5]. On one hand, 3C protease mediates proteolytic processing of the viral polyprotein, releasing capsid and nonstructural proteins critical for viral assembly [4,6]. On the other hand, it cleaves multiple key host innate immune proteins, including porcine cyclic GMP–AMP synthase (pcGAS), porcine gasdermin A (pGSDMA), and porcine Pro-IL-1β, to subvert host innate immune defenses or induce inflammation [7–10]. Consistent with this, inhibition of 3C protease activity impedes viral replication in multiple picornaviruses. For example, rupintrivir, a covalent inhibitor, effectively targets the 3C protease active site in enteroviruses, human rhinovirus type C, foot-and-mouth disease virus (FMDV), and SVV [11,12]. Similarly, the main protease (3-chymotrypsin-like protease, 3CL pro) of severe acute respiratory syndrome coronavirus 2 (SARS-CoV-2) plays a pivotal role in the viral life cycle, confirming its status as an attractive drug target for combating coronavirus disease 2019 (COVID-19) [13]. Simnotrelvir, an orally bioavailable inhibitor of the SARS-CoV-2 3CL pro, covalently inhibits the enzyme with high selectivity [14]. Furthermore, 3C proteases are highly conserved among picornaviruses and exhibit relatively low mutation frequencies [15,16], reinforcing their value as druggable targets.

Traditional antiviral development has emphasized small-molecule covalent protease inhibitors [17]. While these agents may offer favorable oral bioavailability and rationally designed mechanisms, they often suffer from limited selectivity and off-target toxicity [18]. In our own previous screen of 23 cysteine and serine protease inhibitors and 6 additional compounds, Z-VAD (Ome)-FMK was found to significantly suppress SVV 3C protease cleavage activity using a recombinant protein system [19]. In contrast, peptide-based inhibitors are emerging as a highly promising alternative strategy in a competitive or non-competitive mode [20–22]. By mimicking natural substrate cleavage motifs, these peptides bind to the target proteins with specificity through precise engagement of the protease active site, thereby minimizing off-target effects [17,23,24]. Advances in peptide engineering, such as backbone cyclization and non-natural amino acid incorporation, have dramatically enhanced their metabolic stability, membrane permeability, and pharmacological properties [25], which fine-tunes affinity, selectivity, and stability positions to be rationally designed peptide inhibitors as versatile and inherently safer therapeutic agents [26]. Consequently, the unique advantages of peptides offer the compelling and promising approach to develop novel, potent, and selective 3C protease inhibitors with improved therapeutic potential over conventional small molecule approaches. For example, Eberle et al. described all-d-peptides interact with the SARS-CoV-2 3CL pro with high affinity and inhibit its activity, which could be further optimized for the application of the prevention and treatment of COVID-19 [27]. Mp-4D7-pF2, a novel macrocyclic peptide, binds to the 3CL pro sites through a non-covalent mechanism, effectively inhibiting its activity and thereby exerting antiviral activity against SARS-CoV-2 infection [28]. Therefore, developing the peptide inhibitors of 3C protease that feature improved cell membrane permeability and potent antiviral activity would be a promising approach to develop novel therapeutics.

In this study, we rationally designed the decapeptide P5 by integrating key amino acid motifs derived from three distinct 3C protease cleavage sites. P5 potently inhibited 3C protease with negligible cytotoxicity and demonstrated promising activity by effectively blocking 3C-mediated cleavage of multiple host immune proteins. Mechanistically, P5 directly interacted with 3C His48, competing with 3C-substrate binding in a dose-dependent manner. Furthermore, a key mechanistic breakthrough was our finding that P5 rescues cGAS phase separation from 3C protease disruption, thereby restoring downstream type I interferon (IFN-I) induction. Together, these findings establish P5 as a promising peptide therapeutic lead and provide a framework for developing next-generation antiviral inhibitors for the treatment of SVV infections.

## Results

### Rational design of the P5 peptide enables potent inhibition of viral 3C protease

**Fig 1:**
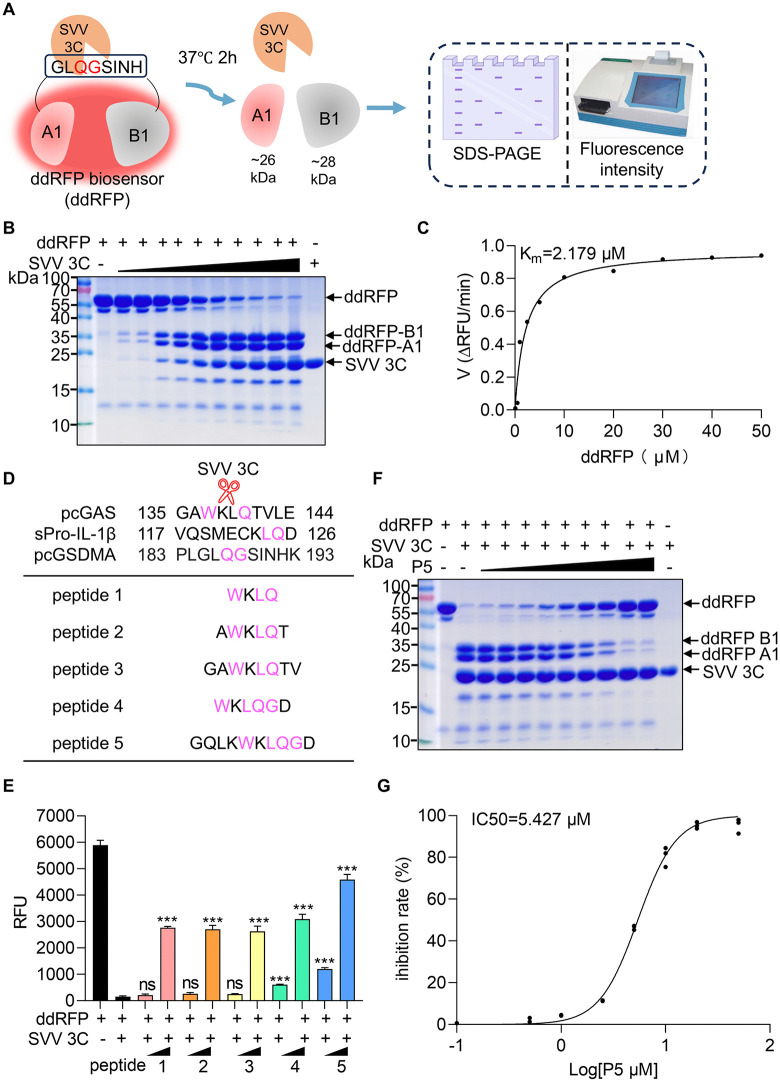
Rational design of P5 peptide enables potent inhibition of SVV 3C protease. **(A)** Schematic of ddRFP biosensor design. RFP-A1 and RFP-B1 domains are linked by GLQGSINH to enable dimerization-dependent fluorescence. 3C protease cleavage at the linker site dissociates domains, leading to fluorescence loss. Protease activity was quantified via SDS-PAGE and fluorescence. Created in BioRender. Yin, **H.** (2026) https://BioRender.com/6excajw **(B)** SDS-PAGE analysis of ddRFP cleavage in a reaction buffer containing 20 μg ddRFP recombinant protein with different dose of purified recombinant protein SVV 3C (0.01, 0.1, 0.5, 1, 2, 5, 10, 15, 20, 25 μg) for 2 h at 37°C *in vitro*. **(C)** Michaelis-Menten plot for the ddRFP biosensor. The Km value is shown. **(D)** Cleavage motif sequence of pcGAS, pGSDMA and sPro-IL-1β of SVV 3C. Display of the designed peptide sequence. Purple sites, the cleavage sites of SVV 3C. **(E)** RFU value analysis assessing the inhibition of the 3C cleavage activity with the designed peptides. The samples containing 20 μg ddRFP protein were mixed with the 10 μg 3C and peptide (0.1 and 10 μM) for 2 h at 37°C. The RFU value was recorded via a microplate reader. **(F)** SDS-PAGE analysis of ddRFP cleavage in a reaction buffer containing 20 μg ddRFP recombinant protein with 10 μg 3C and different concentrations of P5 (0.01-40 μM) for 2 h at 37°C. **(G)** Dose-response curve of P5 against the 3C protease. The IC_50_ value is shown. The samples containing 20 μg ddRFP recombinant protein with 10 μg 3C and different concentrations of P5 (0.01-70 μM) for 2 h at 37°C.

The dimerization-dependent red fluorescent protein (ddRFP) system, which relies on the interaction between a quenched RFP-A1 domain and a nonfluorescent RFP-B1 domain to generate high fluorescence, is a powerful biochemical tool for monitoring protease activity and inhibition [29,30]. Previous work developed a enterovirus 71 (EV71) 3C protease biosensor utilizing the ddRFP system, in which the two RFP domains were linked by a 3C pro cleavage motif (IEALFQG) [31]. Building on this strategy, we constructed a ddRFP biosensor containing RFP-A1 and RFP-B1 domains connected by a 3C protease cleavage motif (GLQGSINH) derived from the cleavage sites of porcine Gasdermin A (pGSDMA) (S1A Fig) [10]. In the presence of 3C protease, this linker between RFP-A1 and RFP-B1 domains was cleaved, releasing the heterodimer and leading to a marked decrease in fluorescence (Fig 1A). Later, the ddRFP biosensor was highly expressed as a soluble protein in *E*.coli cells and purified using Ni-NTA affinity followed by gel filtration chromatography. SDS-PAGE analysis revealed that the purified ddRFP migrated as a single band at the expected molecular mass of 55 kDa, confirming its identity and purity for subsequent screening assays (S1B and S1C Fig). To evaluate substrate activity, ddRFP protein was incubated with increasing amounts of 3C at 37°C. After 2 h, cleavage was observed as a dose-dependent reduction in the full-length ddRFP band, accompanied by the appearance of two new cleavage bands of approximately 26 and 28 kDa (Fig 1B), which was confirmed by measurements showing that ddRFP had a Km of 2.179 μM (Fig 1C).

Our prior studies have shown that SVV 3C protease directly cleaves pGSDMA, cytosolic and nuclear porcine cGAS (pcGAS) and porcine Pro-IL-1β (sPro-IL-1β) [7–10]. Based on the consensus cleavage sequences WQ/QG/LQ of SVV 3C cleavage (S2 Fig), we synthesized a series of peptides derived from sequences within the region of 3C. The first three peptides were designed from the cleavage motif “WKLQ” of pcGAS by 3C protease. These peptides were subsequently extended to form hexapeptide and octapeptide by incorporating the IL-1β cleavage motif “LQ”. The fourth peptide was derived from peptide 1 by incorporating the pGSDMA cleavage site “QG”, while the fifth was designed with a symmetrical repeat of the KLQG motif around a central W residue. These five peptides, designated P1 to P5, were used to analyze the inhibition of SVV 3C activity (Fig 1D). The inhibitory potency of these peptides was assessed using a combination of protein cleavage and kinetic protease assays. While 3C protease alone significantly reduced fluorescence, co-treatment with peptides P1-P5 in the cleavage assay protected ddRFP from cleavage, with P5 exhibiting the strongest inhibitory effect (S3 Fig). Moreover, SVV 3C treatment abolished ddRFP fluorescence, but co-treatment with peptides restored fluorescence in a dose-dependent manner, with P5 showing the highest restoration (Fig 1E). To further investigate the inhibitory efficiency of P5, SDS-PAGE analysis showed that P5 peptide inhibited 3C protease-mediated cleavage of ddRFP in a dose-dependent manner, reducing the production of RFP-A1 (~26 kDa) and RFP-B1 (~28 kDa) fragments (Fig 1F). Furthermore, a protease inhibition assay determined that P5 had a half-maximal inhibitory concentration (IC50) of 5.427 μM against 3C protease, confirming its potent inhibitory effect (Fig 1G). Collectively, these results demonstrate that P5 is a potent inhibitor of 3C protease and a promising candidate for small-molecule inhibitor development.

### Membrane permeability, safety and stability of the P5 peptide

**Fig 2:**
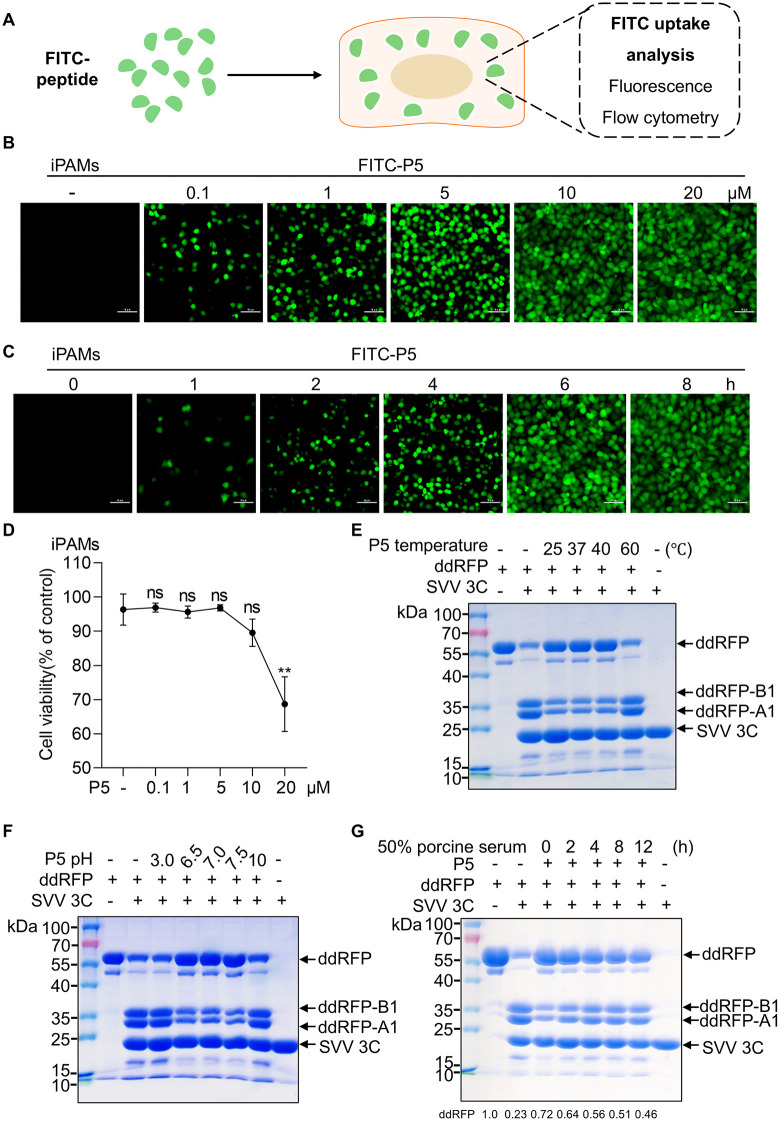
Membrane permeability, safety and stability of the P5 peptide. **(A)** Schematic of the FITC-P5 peptide permeation assay. Cells were incubated with FITC-conjugated P5 peptide at specified concentrations and time points. FITC-positive cells were quantified by fluorescence microscopy and flow cytometry. (**B**) iPAMs were treated with the FITC-peptide (0.1-20 μM) for 10 **h.** Fluorescence microscopy analysis the number of FITC-positive cells. Scale bar, 50 µm. (**C**) iPAMs were treated with the FITC-peptide (10 μM) for 1, 2, 4, 6 and 8 **h.** Fluorescence microscopy analysis the number of FITC-positive cells. Scale bar, 50 µm. (**D**) iPAMs were treated with increase concentrations of P5 peptide for 6 h and cell viability was assessed by MTT assay after P5 peptide treatment. (E, F and **G) (E)** P5 was pre-incubated at different temperatures (25, 37, 40, or 60°C) for 2 **h. (F)** P5 was pre-incubated at different pH values (3, 6.5, 7, 7.5, or 10). **(G)** P5 was incubated with 50% porcine serum at 37°C for 0, 2, 4, 8, or 12 **h.** The treated peptide was then subjected to an *in vitro* cleavage assay in reaction mixtures containing 20 μg recombinant ddRFP substrate, 10 μg 3C protease, and 40 μM P5, followed by incubation at 37°C for 2 h and SDS–PAGE analysis. The ddRFP band intensity was quantified using ImageJ software.

To assess the cell-penetrating efficiency of the P5 peptide, FITC-conjugated P5 was synthesized and incubated with cells, followed by detection of FITC signals (Fig 2A). Immortalized porcine alveolar macrophages (iPAMs) were treated with P5 at concentrations of 0.1, 1, 5, 10 and 20 μM for 10 h, and cellular uptake of P5 was analyzed by fluorescence microscopy and flow cytometry. Fluorescence intensity significantly increased in a dose-dependent manner, peaking at 10 μM (Figs 2B and S4A). These results indicated that P5 achieved optimal cellular penetration at 10 μM. To further investigate the effect of incubation time on P5 penetration, cells were treated with 10 μM P5 for 1, 2, 4, 6, and 8 h. We observed the penetration increased in a time-dependent manner, reaching maximum levels at 6–8 h (Fig 2C). Correspondingly, the proportion of FITC⁺ cells increased, plateauing after 6 h (S4B Fig). Because epithelial cells are a major target cell type for SVV infection and replication, we further evaluated the cell-penetrating efficiency of the P5 peptide in porcine kidney-15 (PK-15) cells, a porcine epithelial cell line. Similar to the results in iPAMs, P5 showed optimal cellular uptake in PK-15 cells at a concentration of 10 μM after 6 h of incubation (S4C and S4D Fig). To assess potential cytotoxicity, iPAMs were treated with varying P5 concentrations for 6 h. No morphological alterations happened at 0.1, 1, 5 and 10 μM compared to the control, but a significant increase in dead cells was noted at 20 μM (S4E Fig). The 3-(4,5-dimethylthiazol-2-yl)-2,5-diphenyltetrazolium bromide (MTT) assay confirmed that P5 had no significant impact cell viability at concentrations up to 10 μM, with toxicity observed at 20 μM (Fig 2D). Collectively, these results demonstrate that P5 exhibits efficient cellular penetration and a favorable safety profile at 10 μM with a 6 h incubation.

Given that peptides often undergo rapid degradation and exhibit limited stability, we next evaluated the stability of P5 under different physicochemical conditions, including variations in temperature and pH. To do this, we used an *in vitro* 3C protease cleavage assay to evaluate the retained inhibitory activity of P5, with ddRFP cleavage serving as a functional readout of peptide stability. To examine thermal stability, P5 was pre-incubated at 25°C, 37°C, 40°C, or 60°C for 2 h before the cleavage assay. SDS-PAGE analysis showed that P5 pre-incubated at 25°C, 37°C, or 40°C effectively inhibited 3C-mediated cleavage of ddRFP, as indicated by reduced generation of the RFP-A1 (~26 kDa) and RFP-B1 (~28 kDa) fragments (Fig 2E). In contrast, this inhibitory activity was completely lost after pre-incubated at 60°C, indicating that P5 retains functional stability within physiological temperature range (37–40°C) (Fig 2E). We then evaluated the pH stability of P5. The peptide maintained its inhibitory activity under near-neutral conditions (pH 6.5-7.5), whereas the inhibitory effect was abolished under strongly acidic or alkaline conditions (Fig 2F). These results demonstrate that P5 remains functionally stable under physiologically relevant temperature and pH conditions. To further assess its biological half-life, P5 was incubated in 50% porcine serum at 37°C for 0, 2, 4, 8, and 12 h before the cleavage assay. The inhibitory activity of P5 was largely maintained for up to 8 h, with approximately 50% of its inhibitory activity retained after 8 h of incubation (Fig 2G). Taken together, these results suggest that P5 has a functional half-life of approximately 8 h and exhibits favorable stability under simulated physiological conditions.

### Inhibitory activity of P5 against SVV 3C protease-mediated cleavage

**Fig 3:**
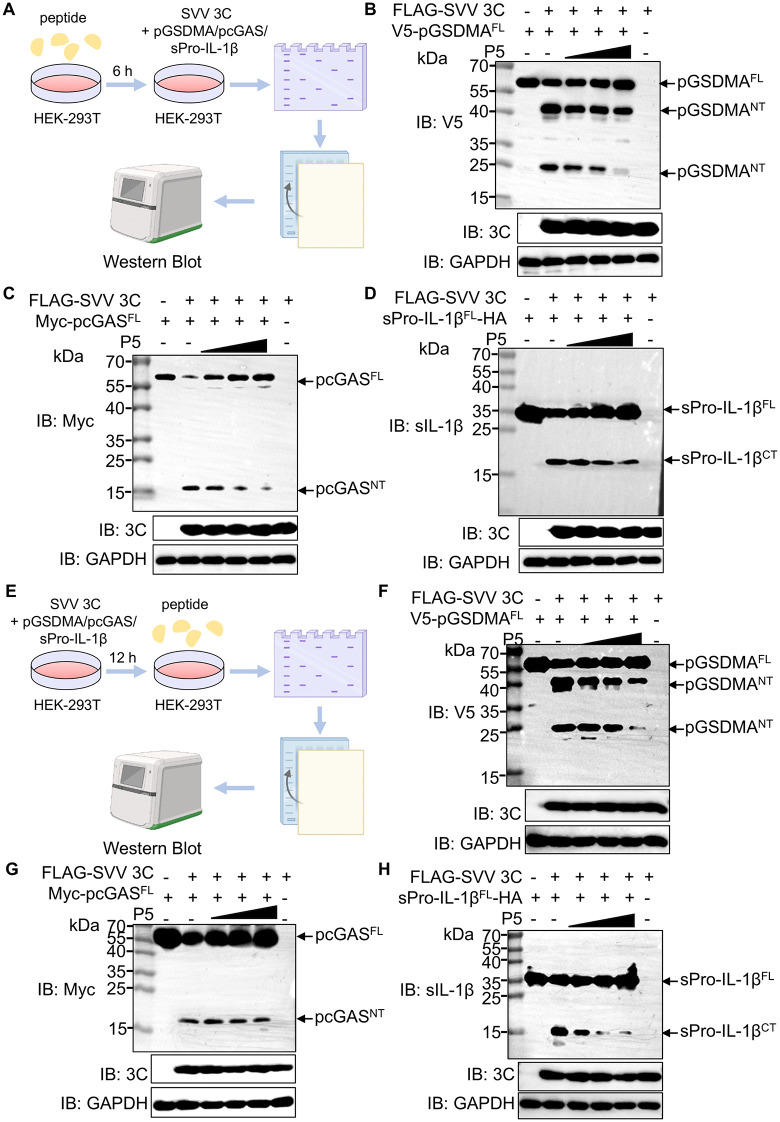
Specific inhibitory activity of P5 against SVV 3C protease-mediated cleavage. **(A)** Schematic illustration of the cell-based assay in which cells were pre-treated with P5 before expression of SVV 3C and its substrates. Created in BioRender. Yin, **H.** (2026) https://BioRender.com/xxq1kgh (**B, C and** **D)** HEK-293T cells were pre-treated with different concentrations of P5 peptide for 6 h and then co-transfected with a plasmid encoding FLAG-SVV 3C together with plasmids encoding V5-pGSDMA, Myc-pcGAS or sPro-IL-1β-HA for 24 **h.** Western blot was used to analyze the P5 (0.1, 1, 10 μM) effects on pGSDMA (B), pcGAS (C) and sPro-IL-1β (D) cleavage. **(E)** Schematic illustration of the cell-based assay in which P5 was added after co-expression of SVV 3C and its substrates. Created in BioRender. Yin, **H.** (2026) https://BioRender.com/bnrq4yd. (**F, G and** **H)** HEK-293T cells were co-transfected with a plasmid encoding FLAG-SVV 3C together with plasmids encoding V5-pGSDMA, Myc-pcGAS or sPro-IL-1β-HA for 18 h, followed by treatment with different concentrations of P5 peptide for 6 h**.** Western blot was used to analyze the P5 (0.1, 1, 10 μM) effects on pGSDMA (F), pcGAS (G) and sPro-IL-1β (H) cleavage.

Previous studies have shown that SVV 3C protease directly cleaves pGSDMA, pcGAS and sPro-IL-1β [7–10]. Given P5’s potent inhibition of SVV 3C activity, we assessed its inhibitory effects on these substrates. Recombinant SVV 3C protein was incubated with pGSDMA^FL^, pcGAS^FL^, sPro-IL-1β^FL^ in the presence of increasing P5 concentrations at 37°C for 2 h, followed by SDS-PAGE analysis (S5A Fig). In the absence of P5, recombinant SVV 3C protein reduced bands of the full-length proteins and generated cleavage products. However, increasing P5 concentrations restored the bands of the full-length proteins and reduced cleavage fragments, confirmed P5-mediated inhibition of 3C proteolytic activity (S5B–S5D Fig). To evaluate P5-mediated intracellular inhibition of 3C protease, HEK-293T cells were pre-treated with P5 for 6 h, followed co-expression of FLAG-tagged SVV 3C along with V5-pGSDMA, Myc-pcGAS, or HA-tagged sPro-IL-1β, respectively, and subsequently detected by western blotting (Fig 3A). Consistent with prior reports, co-expression with 3C protease produced clear cleavage bands of pGSDMA (NT, ~ 40 and 25 kDa), pcGAS (NT, ~ 15 kDa) and sPro-IL-1β (CT, ~ 15 kDa). P5 inhibited SVV 3C cleavage in a dose-dependent manner (Fig 3B–3D), confirming its ability to suppress SVV 3C-mediated proteolysis in cells. Because, in infected cells, SVV 3C protease may already be associated with its host substrates before treatment, we next performed a post-treatment experiment to better mimic the therapeutic setting. HEK-293T cells were first co-transfected with SVV 3C and the indicated substrates, followed by addition of P5 (Fig 3E). Under these conditions, SVV 3C protease again generated the expected cleavage products of pGSDMA (NT, ~ 40 and 25 kDa), pcGAS (NT, ~ 15 kDa), and sPro–IL-1β (CT, ~ 15 kDa). Importantly, P5 still inhibited SVV 3C-mediated cleavage of all three substrates in a dose-dependent manner (Fig 3F–3H), further supporting its ability to block 3C protease activity in cells and highlighting its potential as a therapeutic inhibitor.

### P5 binds the catalytic pocket of SVV 3C protease

**Fig 4:**
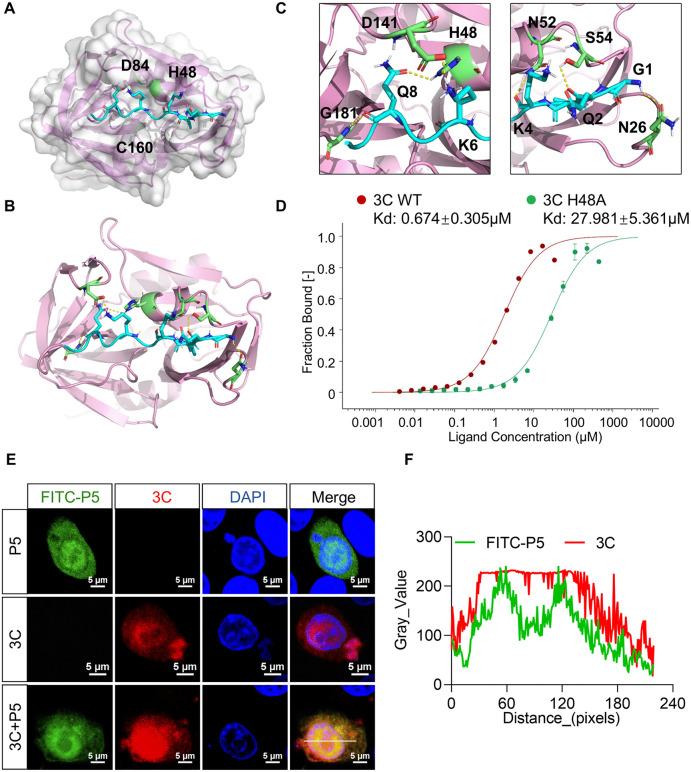
P5 binds the catalytic pocket of SVV 3C protease. **(A)** Molecular docking of peptide P5 with SVV 3C using HADDOCK2.4 on the website. **(B)** The H-bonds interaction between 3C (pink) and P5 (blue) were indicated by dashed lines (yellow). **(C)** Detailed H-bonds sites of SVV 3C (pink) and P5, in which SVV 3C (G181, D141, H48, N52, S54, N26) was marked in green and P5 was marked in blue. **(D)** Binding affinities of SVV 3C wild-type (WT) and H48A mutant proteins with FITC-P5 peptide measured by MST. **(E and F)** HeLa cells were treated with FITC-P5 and transfected with SVV 3C for 24 **h.** Cells were immunostained with an anti-FLAG antibody (red) and stained with DAPI. **(E)** Co-localization of P5 with the 3C was analyzed using confocal microscopy. Scale bars = 5 μm. **(F)** The co-localization was quantified using ImageJ software.

To elucidate the mechanism by which P5 interferes with the proteolytic function of SVV 3C, we utilized HADDOCK2.4 on the website to predict the complex structure of 3C bound to P5. The structural model revealed that P5 peptide was effectively embedded into a positively charged pocket near the enzymatic active site of SVV 3C, formed by residues including His 48 (Fig 4A). Notably, binding of P5 to 3C was primarily mediated by seven hydrogen bonds involving SVV 3C residues Gly 181, Asp 141, His 48, Asn 52, Ser 54, and Asn 26 (Fig 4B). Importantly, the key P5 residue K6 formed a hydrogen bond with His 48 of 3C, enabling P5 to occupy the active site interface (Fig 4C). To quantify the binding affinity of P5 peptide and 3C protease, we conducted Microscale Thermophoresis (MST) assays by testing the interaction of FITC-labeled P5 with 3C wild-type (WT) and H48A mutant proteins, respectively. The results revealed a binding affinity of 0.674 μM for 3C WT with P5, compared to 27.981 μM for 3C H48A with P5, indicating the critical role of 3C His48 binding to P5 (Fig 4D). To further determine whether P5 interacts with SVV 3C in cells, HeLa cells were transfected with SVV 3C for 18 h followed by treatment with P5 for 6 h. Fluorescence microscopy revealed clear co-localization between P5 and SVV 3C in cells (Fig 4E and 4F), supporting the intracellular interaction between the peptide and the protease. Collectively, these results indicate that P5 directly binds SVV 3C protease and likely inhibits its activity by occupying the catalytic pocket and interfering with substrate access.

### P5 restores cGAS-DNA phase separation by inhibiting 3C protease

**Fig 5:**
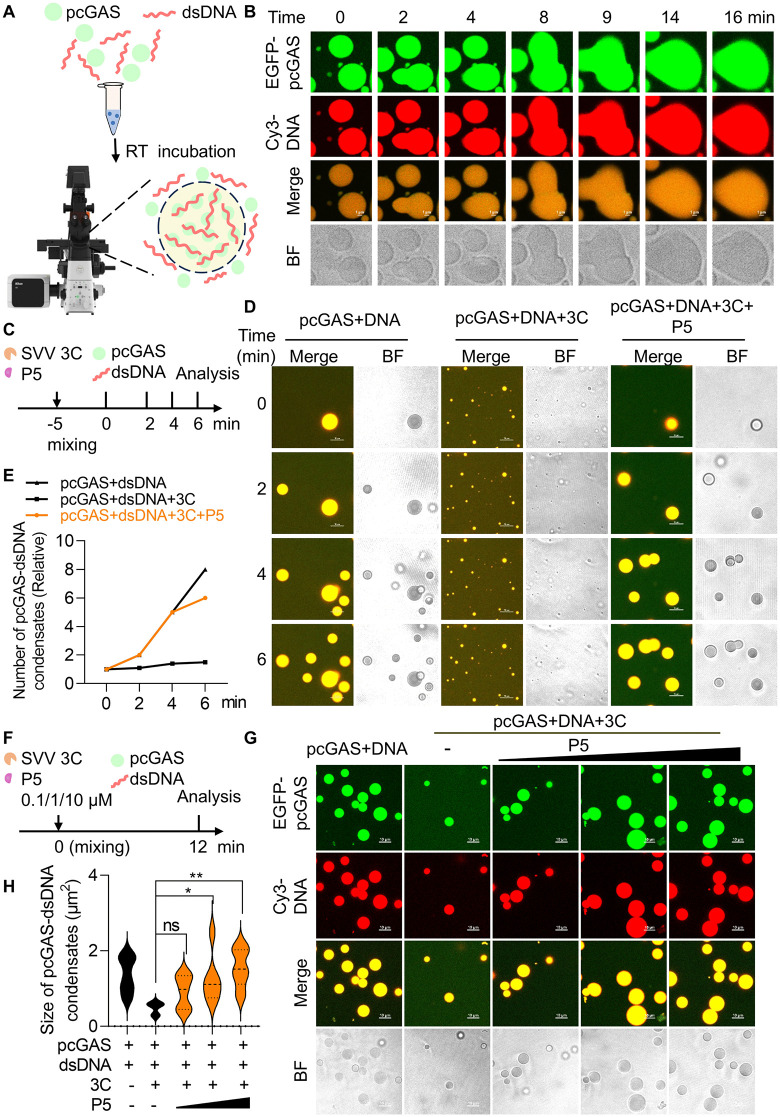
P5 restores cGAS-DNA phase separation by inhibiting 3C protease. **(A)** Schematic of porcine cGAS-DNA interactions driving liquid-liquid phase separation (LLPS). Samples containing 10 μM EGFP-cGAS were mixed with 5 μM Cy3-dsDNA and incubated at 25°C for 5 min. Created in BioRender. Yin, **H.** (2026) https://BioRender.com/lsdrguy. **(B)** Time-lapse imaging of pcGAS-DNA phase separation. Scale bar, 1 μm. **(C)** Schematic of P5 peptide time effect on disruption of cGAS-DNA phase separation by 3C. The samples containing 10 μM EGFP-pcGAS was mixed with the 5 μM Cy3-dsDNA, 10 μM 3C and 10 μM P5 for 5 min, then the form of cGAS-DNA dropts were detected via fluorescence at 2, 4 and 6 min. **(D and E)** Time-lapse imaging of P5-restored cGAS-DNA LLPS. Scale bars, 10 μm. The relative number of cGAS-DNA condensates were shown in **E. (F)** Schematic of dose-dependent P5 protection against 3C-mediated LLPS disruption. Samples containing 10 μM EGFP-cGAS, 5 μM Cy3-dsDNA, 10 μM 3C protease, and P5 (0.1, 1, or 10 μM) were incubated for 12 min. Condensate formation was analyzed by fluorescence microscopy. **(G and H)** Time-lapse imaging of cGAS-DNA LLPS under varying P5 concentrations. Scale bars, 10 μm. The size of cGAS-DNA condensates was shown in H**.** Data are represented as means ± SD from three biological replicates. ns, no significance, *p < 0.05, **p < 0.01, Student’s t-test.

We previously reported that SVV 3C cleaves cytosolic and nuclear pcGAS, disrupting IFN-I signaling for immune evasion [7,8]. Furthermore, we established a phase separation system for pcGAS with dsDNA and demonstrated that circovirus Rep disrupts cGAS oligomerization and phase separation. Under physiological conditions (150 mM NaCl), pcGAS-DNA condensates form gel-like structures, unlike the liquid droplets observed with human cGAS [32]. To optimize these conditions, we added 10% Ficoll to disrupt the macromolecular crowding, promoting a phase transition from gel-like to liquid droplet condensates. This was accompanied by increased fluorescence intensity and larger equivalent diameter (Figs 5A and 5B, S6A) [33,34]. Fluorescence recovery after photobleaching (FRAP) assays confirmed the mobility of pcGAS and DNA within these liquid droplets, as fluorescence efficiently recovered after photobleaching (S6B and S6C Fig). Given P5’s competitive binding to SVV 3C protease, we hypothesized that P5 could restore cGAS-DNA phase separation by inhibiting 3C-mediated cleavage. To test this, we incubated cGAS, 45 bp dsDNA, SVV 3C with P5 peptide for 5 min, followed by analysis at 2, 4, and 6 minutes (Fig 5C). Mixing pcGAS with 45-bp dsDNA rapidly induced liquid-liquid phase separation (LLPS) with enhanced molecular diffusion (first column in Fig 5D). Addition of 3C protease cleaved pcGAS, disrupting DNA binding and forming small, self-aggregated droplets in a time-dependent manner (middle column in Fig 5D). Notably, P5 peptide restored pcGAS-dsDNA liquid droplets by inhibiting 3C protease cleavage (last column in Fig 5D). Quantification confirmed that P5 significantly increased the number of pcGAS-dsDNA condensates (Fig 5E). Consistent with these imaging results, western blot analysis showed that SVV 3C cleaved EGFP-pcGAS (~80 kDa) to generate a ~ 40 kDa EGFP-N-terminal (EGFP tag 25 kDa plus NT 15 kDa), and this cleavage was significantly inhibited by P5, indicating that P5 blocks SVV 3C-mediated cGAS cleavage (S7A Fig). To evaluate the dose-dependent effects of P5 on pcGAS-DNA phase separation, we incubated pcGAS, 45-bp dsDNA, and SVV 3C with increasing P5 concentrations for 12 min and analyzed resulting condensates by fluorescence microscopy (Fig 5F). Consistent with time-course experiments, 3C protease reduced the number of pcGAS-DNA liquid droplets, while P5 dose-dependently restored both the number and size of pcGAS-DNA condensates, suggesting inhibition of 3C-mediated cleavage (Fig 5G and 5H). In parallel, cleavage of pcGAS was also inhibited by P5 in a dose-dependent manner, as shown by the reduction in the N-terminal cleavage fragments and preservation of the full-length pcGAS band (S7B Fig). Taken together, P5 peptide suppresses SVV 3C-mediated cleavage of cGAS, thereby restoring cGAS-DNA phase separation.

### P5 blocks SVV 3C enzymatic activity to restore IFN-I production

**Fig 6:**
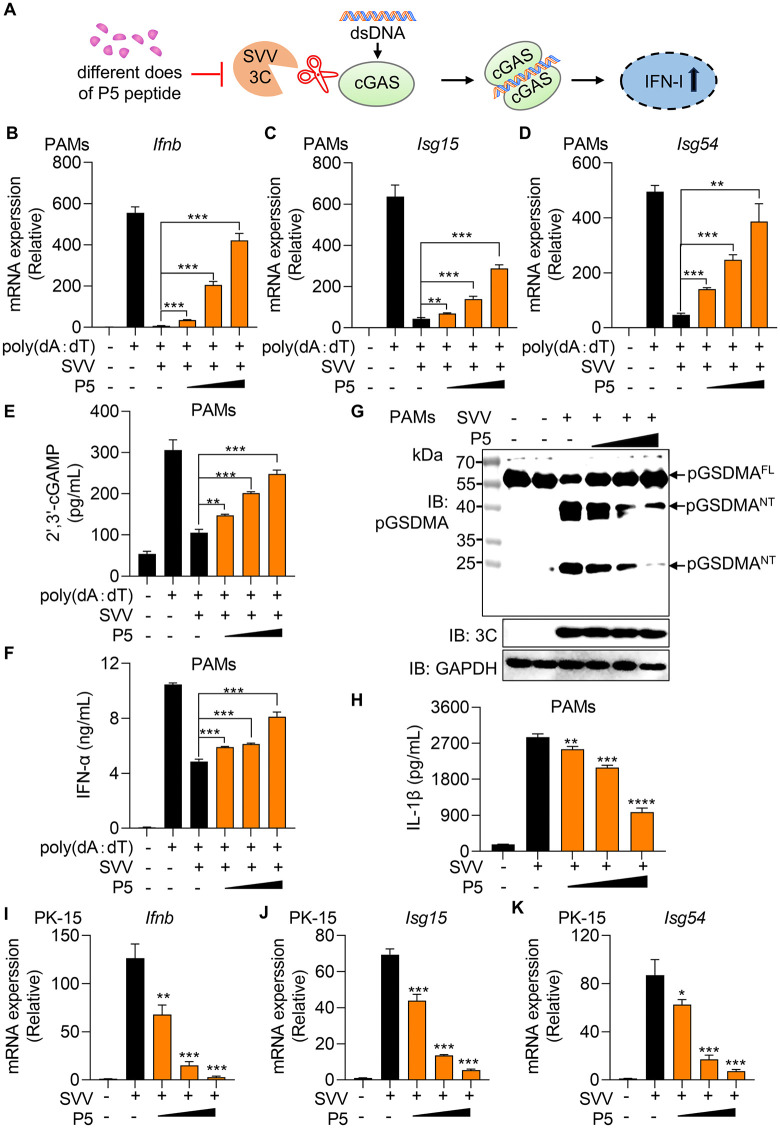
P5 blocks SVV 3C activity to restore IFN-I production. **(A)** Schematic of P5-mediated regulation of IFN-I production. Upon dsDNA stimulation, cGAS dimerization induces IFN-I expression. SVV 3C protease cleaves cGAS, dissociating its N/C-terminal domains and suppressing IFN-I. P5 competitively binds to 3C protease, preventing cGAS cleavage and restoring IFN-I production. (**B**, **C** **and** **D)** PAMs were treated with increasing does of P5 for 6 h, then infected with SVV (MOI = 1). After 12 h, cells were transfected with poly (dA:dT) (1 µg/ml) for 12 **h.** RT-PCR analysis of *Ifnb* **(B)**, *Isg15* (C) and *Isg54* (D) mRNA expression. **(E and F)** ELISA analysis of the production of 2′3′-cGAMP (E) and IFN-α (F) in PAMs treated as above. **(G)** Endogenous pGSDMA cleavage in PAMs treated as described above was analyzed by western blot. **(H)** Secreted IL-1β levels in PAM culture supernatants were measured by ELISA. (**I**, **J** **and** **K)** PK-15 cells were treated with increasing concentrations of P5 for 6 h and then infected with SVV (MOI = 1). After 12 h, cells were harvested for RNA extraction and RT-PCR analysis of *Ifnb* (I) and downstream *Isg15* (J) and *Isg54* (K) mRNA expression. Data are represented as means ± SD from three biological replicates. ns, no significance, **p < 0.01, ***p < 0.001, ****p < 0.0001, Student’s t-test.

LLPS of cGAS-DNA promotes 2′,3′-cGAMP production and thereby drives IFN-I induction [33]. We previously reported that SVV 3C protease cleaves cGAS, disrupting mitochondrial DNA-mediated IFN-I production [7]. Given that P5 binds SVV 3C and inhibits its cleavage of pcGAS, thereby restoring cGAS-DNA phase separation, we hypothesized that P5 could also rescue IFN-I responses (Fig 6A). To test this, iPAMs were transfected with increasing amounts of SVV 3C and then stimulated with poly (dA:dT). The mRNA levels of *Ifnb*, *Isg54* and *Isg56* were significantly reduced compared to the control group, confirming the inhibition of IFN-I by SVV 3C (S8 Fig). Next, we examined whether P5 could reverse this effect. iPAMs and PK-15 cells were pre-treated with increasing P5 concentrations for 6 h, followed by SVV 3C overexpression for 12 h and then poly(dA:dT) stimulation. The mRNA expression of *Ifnb, Isg15 and Isg54* increased in a dose-dependent manner, peaking at 10 μM (S9 Fig). To validate these results under physiological conditions, primary porcine alveolar macrophages (PAMs) isolated from alveolar lavage fluid were pre-treated with 0.1, 1, or 10 μM P5 for 6 h, infected with SVV (MOI = 0.1) for 12 h, and stimulated with poly(dA:dT) for 12 h. Consistently, *Ifnb*, *Isg15*, and *Isg54* mRNA levels increased in a dose-dependent manner in PAMs (Fig 6B–6D). Furthermore, P5 treatment enhanced 2′,3′-cGAMP production and IFN-α secretion in a dose-dependent manner, confirming inhibition of SVV 3C activity by P5 (Fig 6E and 6F). To assess whether P5 inhibits SVV 3C-mediated substrate cleavage under infection conditions, we generated custom polyclonal antibodies against porcine proteins by immunizing mice with purified recombinant antigens. Among these, we successfully obtained a high-quality antibody against pGSDMA. Using this antibody, we examined the endogenous expression of pGSDMA in P5-treated, SVV-infected PAMs. Consistent with our observations, immunoblot analysis showed that SVV 3C protease cleaved pGSDMA into two distinct fragments. Importantly, this cleavage was significantly inhibited by P5 in a dose-dependent manner, indicating effective suppression of 3C protease activity (Fig 6G). IL-1β is a key inflammatory mediator that plays critical roles in host defense and pathological inflammation. We previously reported that SVV 3C protease functions as an unconventional IL-1β–converting enzyme (ICE) by directly cleaving and processing swine Pro–IL-1β [9]. The resulting cleaved IL-1β is biologically active and functionally comparable to mature IL-1β, thereby affecting host inflammatory responses. To determine whether P5 affects this process, we measured the levels of secreted IL-1β (sIL-1β) in the supernatant of SVV-infected macrophages treated with P5. ELISA showed sIL-1β levels were markedly reduced in the P5-treated group compared with the SVV-only group (Fig 6H), suggesting that P5 inhibits SVV 3C-mediated cleavage of sPro–IL-1β.

In addition, we previously showed that SVV infection induces the release of mitochondrial DNA (mtDNA) into the cytosol, which activates IFN-I induction [7]. Based on thess results, we further hypothesized that P5 could restore IFN-I responses suppressed during SVV infection. To test this hypothesis, we measured the mRNA levels of *Ifnb*, *Isg15* and *Isg54* in SVV-infected PK-15 cells treated with P5. Quantitative RT-PCR analysis showed that treatment with P5 significantly increased the mRNA levels of *Ifnb*, *Isg15*, and *Isg54* compared with SVV infection alone (Fig 6I–6K), indicating that P5 partially restores IFN-I induction during infection by inhibiting SVV 3C activity. In addition to cGAS cleavage, SVV also suppresses the RIG-I antiviral signaling pathway to attenuate the IFN-I response [35]. To further evaluate whether P5 influences the RIG-I pathway, we examined its effect on SVV 3C-mediated inhibition of RIG-I signaling. The iPAMs were pre-treated with increasing concentrations of P5, followed by overexpression of SVV 3C for 12 h and subsequent stimulation with poly(I:C). Quantitative RT-PCR analysis showed that the mRNA levels of *Ifnb*, *Isg15*, and *Isg54* increased in a dose-dependent manner upon P5 treatment, (S10A–S10C Fig), indicating that P5 also counteracts SVV 3C-mediated suppression of the RIG-I antiviral signaling pathway. Collectively, these results demonstrate that P5 significantly blocks SVV 3C protease activity, restores cGAS- and RIG-I-dependent antiviral signaling, and promotes recovery of IFN-I responses during SVV infection.

### Antiviral activity of P5 peptide against SVV replication

**Fig 7:**
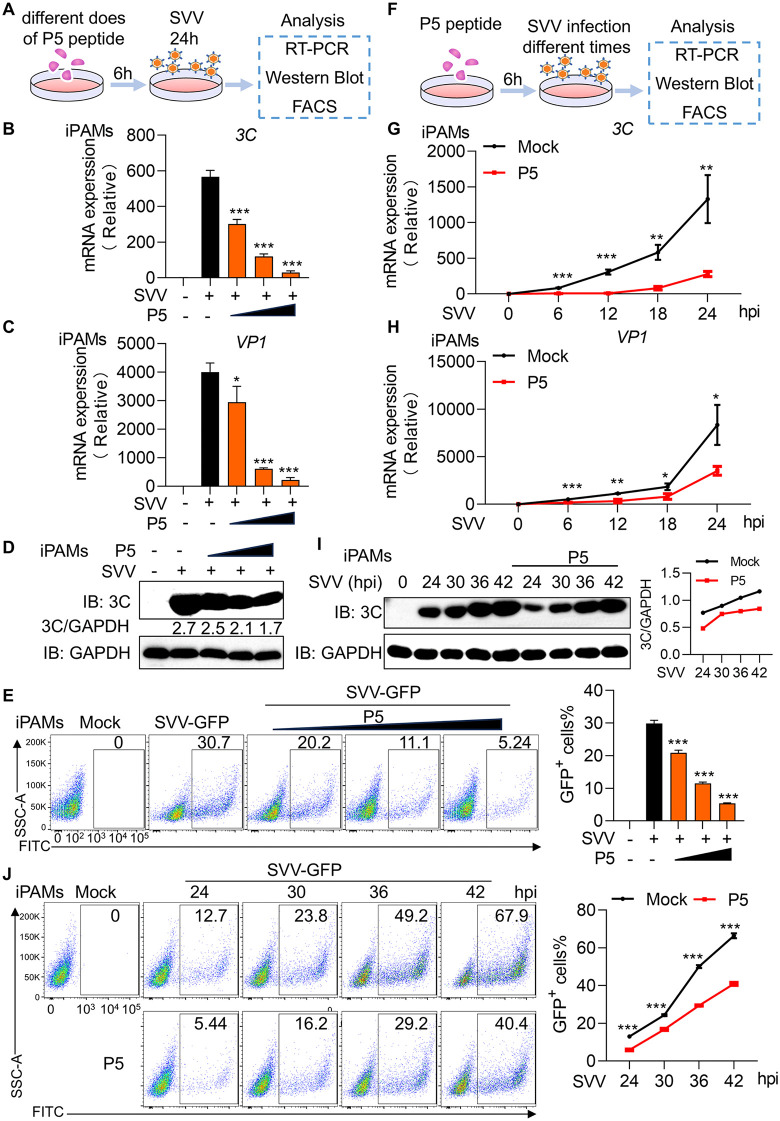
Antiviral activity of P5 peptide against SVV replication. **(A)** Schematic of P5 concentration-dependent effects on SVV replication. iPAMs were pretreated with increasing P5 amounts for 6 h, followed by SVV infection (MOI = 1) for 24 **h. (B and C)** Cells were harvested for RNA extraction and RT-PCR detection of SVV *3C* (B) and *VP1*(C) expression. **(D)** Western blot analysis of SVV 3C protein expression. **(E)** Flow cytometry analysis of GFP⁺ cells after SVV-GFP infection (MOI = 1). Statistical results are shown on the right. **(F)** Schematic of time-dependent P5 effects on SVV replication. iPAMs were pre-treated with P5 (10 μM) for 6 h and infected with SVV (MOI = 0.1) at indicated time points. **(G and H)** RT-PCR analysis of SVV *3C* (G) and *VP1* (H) mRNA expression. **(I)** Western blot analysis of SVV 3C protein expression. **(J)** Flow cytometry quantification of GFP⁺ cells after SVV-GFP infection (MOI = 0.1). Statistical results are shown on the right. Data are represented as means ± SD from three biological replicates. *p < 0.05, **p < 0.01, ***p < 0.001, Student’s t-test.

To investigate the antiviral activity of P5 peptide, iPAMs were pre-treated with increasing P5 concentrations for 6 h and then infected with SVV (MOI = 0.1) for 24 h. Viral replication was assessed by RT-PCR, western blot and flow cytometry (FACS) (Fig 7A). P5 treatment significantly reduced SVV *3C* and *VP1* gene copy numbers in a dose-dependent manner compared to the control (Fig 7B and 7C). Similarly, SVV 3C protein levels were attenuated following P5 treatment (Fig 7D). FACS analysis of SVV-GFP-infected cells revealed a significant reduction in GFP^+^ cells with P5 treatment (Fig 7E). To examine the temporal dynamics of P5′s antiviral effects, iPAMs were pre-treated with P5 for 6 h, and then infected with SVV (MOI = 0.1) for varying durations (Fig 7F). P5 treatment significantly suppressed SVV mRNA levels across different time points (Fig 7G and 7H). Consistently, P5 treatment significantly reduced SVV 3C protein expression and the proportion of GFP^+^ cells, as confirmed by western blot and FACS analysis, respectively (Fig 7I and 7J). Taken together, these results confirm that P5 significantly inhibits SVV replication by disrupting 3C protease cleavage activity.

**Fig 8:**
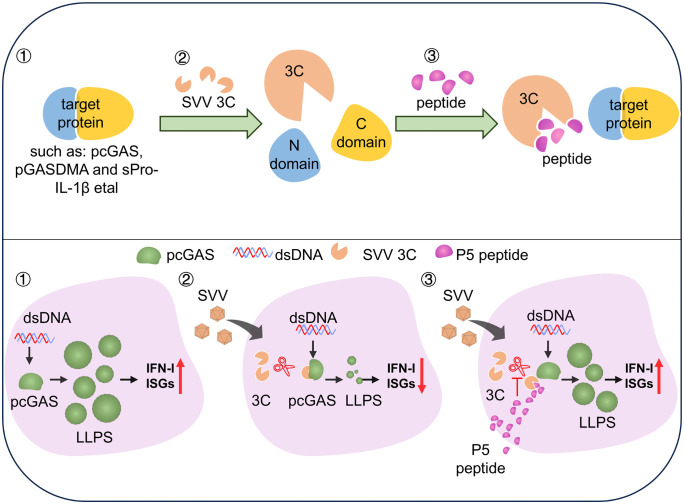
Functional model for P5-dependent 3C inhibition. The P5 peptide effectively inhibits SVV 3C protease-mediated cleavage of target proteins, preserving their immune function. Specifically, P5 counteracts 3C-mediated disruption of cGAS-DNA liquid-liquid phase separation (LLPS), restoring IFN-I production and potently inhibiting SVV replication.

Collectively, these results demonstrate that the peptide inhibitor P5 binds SVV 3C, suppressing its replication and cleavage of innate immune proteins, particularly by restoring cGAS-DNA phase separation to enhance IFN-I production (Fig 8).

## Discussion

SVV causes vesicular disease in swine [15,16], with its 3C protease playing a crucial role in cleaving viral polyprotein into mature viral proteins for assembly and cleaving multiple immune proteins to evade innate immunity [6,36,37]. Peptidomimetics, such as those mimicking the ALFQ substrate, have shown efficacy in inhibiting the enterovirus 71 (EV71) 3C protease [38,39]. Fluorescence resonance energy transfer (FRET) assays are widely used to screen and evaluate protease inhibitors [40,41]. However, peptide substrates often suffer from inherent limitations including instability, high production costs, and low emission wavelengths [40]. To address these challenges, Ye et al. developed a genetically encoded EV71 3Cpro biosensor based on a ddRFP system, offering a robust biochemical tool for rapid assessment of anti-enterovirus drugs [31]. This ddRFP biosensor assay provides a faithful avenue for rapid screening and evaluation of inhibitors against SARS-CoV-2 [29,30,42]. In this study, we adapted this system to engineer a substrate specifically suited for SVV 3C protease, facilitating more effective drug screening. We engineered a ddRFP biosensor incorporating the 3C-mediated pGSDMA cleavage motif as a linker, expressed in *E*. coli. The SVV 3C protease cleaved this ddRFP protein in a dose-dependent manner, with a Km of 2.179 µM, confirming efficient hydrolysis and establishing the ddRFP system as a reliable platform for studying 3C protease activity.

Using a rational substrate mimicry approach, we designed peptide inhibitors targeting SVV 3C protease based on conserved cleavage recognition motifs within host immune proteins, including pGSDMA, pcGAS and sPro-IL-1β. In the functional ddRFP-based assays, the P5 peptide exhibited potent inhibition of 3C protease, with an IC50 value of 5.427 μM. The exceptional efficacy of P5 likely stems from its strategic incorporation of three critical cleavage recognition motifs-LQ, QG, and WQ, derived from key 3C substrate sites. This multi-motif architecture enables P5 to function as a potent competitive inhibitor, effectively blocking 3C protease activity across diverse cleavage substrates. Notably, P5 broadly inhibited 3C-mediated cleavage of pGSDMA, pcGAS, and sPro-IL-1β in both recombinant protein and cellular assays, counteracting viral immune evasion. Structural modeling further indicated that P5 forms a hydrogen bond with His48, a critical residue in the 3C active site, thereby interfering with enzymatic function. Supporting this mechanism, mutation of His48 markedly weakened P5 binding, and confocal microscopy confirmed co-localization of P5 with SVV 3C in cells. Together, these data support a model in which P5 competes with host substrates for binding to SVV 3C, resulting in loss of protease activity.

Peptide-based therapeutics often face challenges related to rapid degradation and limited metabolic stability, which can restrict their *in vivo* utility. In this study, we systematically evaluated the stability of P5 under physiologically relevant conditions. Our results show that P5 retains functional activity across temperatures and pH ranges consistent with normal physiological environments. Notably, P5 maintained its inhibitory activity against SVV 3C protease for up to 8 h after incubation in serum, corresponding to a functional half-life of approximately 8 h. This stability in serum, together with resistance to temperature and pH variations, suggests that P5 possesses favorable biochemical properties for potential *in vivo* application. In addition, P5 achieved highly cellular uptake efficiency at 6 h post-treatment with 10 μM without compromising cell viability, underscoring its therapeutic safety. This favorable uptake, safety profile, and good stability are essential prerequisites for future translational development.

The DNA sensor cGAS was first shown by Dr. Chen′s laboratory to form LLPS condensates with dsDNA, thereby enhancing 2′,3′-cGAMP production and activating IFN-I signaling [33]. Notably, cGAS phase separation exhibits species-specific differences [34]. Under varying salt concentrations, porcine cGAS predominantly forms gel-like assemblies rather than liquid droplets. However, addition of Ficoll promotes the formation of droplet-like pcGAS–DNA condensates characteristic of LLPS. SVV 3C cleavage of pcGAS impairs the pcGAS-DNA condensates formation and IFN-I production [7]. Remarkably, P5 treatment uniquely rescued 3C-mediated disruption of cGAS-DNA phase separation in a time and dose dependent manner, demonstrating its potential as a novel peptide inhibitor for picornavirus clearance. Until now, only a limited number of agents, such as spermine, oleic acid and acetaldehyde, are known to modulate cGAS phase separation [43–46], while agents like XQ2B (cyclopeptide inhibitors) and XL-3158 represents the specific cGAS inhibitor targeting phase separation [47,48]. Mechanistically, P5 uniquely prevents 3C-mediated cleavage of cGAS, enabling cGAS phase separation. Functionally, P5 treatment restored cGAS phase separation and rescued downstream IFN-I mRNA and protein expression in cells. Beyond cGAS, P5 also inhibited SVV 3C-mediated cleavage of pGSDMA and sPro–IL-1β in PAMs. Furthermore, P5 counteracted the degradation of RIG-I induced by SVV 3C, restoring the expression of IFN-I and interferon-stimulated genes (ISGs). Because IFN-I signaling constitutes a critical innate immune barrier that restricts viral replication through induction of hundreds of ISGs, these findings provide a mechanistic explanation for the antiviral effects of P5. Consistently, P5 treatment suppressed SVV replication in a dose and time dependent manner, confirming its potent antiviral activity.

Peptides have significantly advanced biological and chemical sciences [22,49,50]. For instance, rupintrivir (AG-7088), a peptide-based inhibitor with an electrophilic ethyl propenoate Michael acceptor, targets human rhinovirus 3C protease [51]. Similarly, insulin, a 51-amino-acid peptide, was the first commercially available peptide drug in 1923, with recombinant human insulin introduced in 1982 [52,53]. These precedents highlight the tremendous potential of peptide for selective molecular recognition and clinical applications, and support the development of P5 as a candidate antiviral agent against SVV infection. Compared with conventional small-molecule drugs, peptide-based therapeutics often offer several advantages, including high specificity, favorable safety profiles, good tolerability, and reduced risk of long-term accumulation *in vivo*. In this context, P5 represents a promising lead compound for combating SVV infection. Future *in vivo* studies will be necessary to evaluate its therapeutic efficacy and pharmacological properties in pig models.

In summary, our study reveals that P5 enhances host innate immunity by inhibiting SVV 3C protease activity. Its rational design, incorporating key cleavage motifs, enables potent inhibition of 3C protease, providing a powerful tool against SVV infection and a foundation for developing peptide-based antivirals.

## Materials and methods

### Ethics statement

Porcine Alveolar Macrophages (PAMs) were isolated following the protocols, which were approved by the Committee on the Ethics of Animal Experiments of the Harbin Veterinary Research Institute (HVRI) of the Chinese Academy of Agricultural Sciences (CAAS) and the Animal Ethics Committee of Heilongjiang Province, China. The license number associated with this research protocol was 231017–01-GR.

### Cells and viruses

HEK-293T cells (Human embryonic kidney cells), PK-15 cells (Porcine kidney-15), HeLa cells, and iPAMs (Immortalized porcine alveolar macrophages) were cultured in Dulbecco′s Modified Eagle Medium (DMEM) (MACGENE, #CM10013) containing 10% fetal bovine serum (FBS) (PlantChemMed, #PC-00001) at 37°C with 5% CO_2_, whereas primary porcine alveolar macrophages (PAMs) from 1-mo-old specific pathogen-free (SPF) piglets were maintained in Roswell park memorial institute (RPMI)1640 medium. All cells were incubated at 37°C under 5% CO2 and analyzed by Falcon S400, Intelligent cell imaging and analysis system (Alicelligent Technologies). The Seneca Valley Virus (SVV) and SVV-GFP virus were stored in our laboratory.

### Antibodies

The following antibodies were used in this study. Mouse anti-FLAG MAb (#F1804) was obtained from Sigma-Aldrich (MO, USA). Rabbit anti-GAPDH pAb (#10494–1-AP), rabbit anti-HA pAb (#51064–2-AP), mouse anti-GFP monoclonal antibody (mAb) (#66002–1-Ig), mouse anti-Myc mAb (#10828–1-AP) and CoraLite594-conjugated Goat Anti-Mouse IgG (SA00013–3) were purchased from Proteintech Group (Chicago, IL). Rabbit anti-V5 monoclonal antibody (mAb) (#13202S), mouse anti IgG HRP-linked antibody and anti-rabbit IgG HRP-linked antibody were purchased from Cell Signaling Technology (Beverly, MA). Mouse anti-SVV 3C polyclonal Ab and mouse anti-sIL-1β polyclonal Ab were prepared by our laboratory.

### Plasmids and recombinant proteins

The ddRFP biosensor (ddRFP), containing RFP-A1 (GenBank: JN381545) and RFP-B1 (GenBank: JN381546) domains with a polypeptide linker (GLQGSINH), was cloned into His6-pET28a vector. The pcDNA3.1-FLAG-SVV 3C, pcDNA3.1-V5-pGSDMA, pcDNA3.1-Myc-pcGAS and pcDNA3.1-sPro-IL-1β-HA were stored in our laboratory. The recombinant protein full-length pGSDMA, pcGAS, sPro-IL-1β, SVV 3C WT and H48A protein and EGFP-pcGAS were stored in our laboratory.

### Peptides

Peptides P1 to P5 and FITC-labelled P5 peptide were synthesized by Nanjing TG peptide Co (Nanjing, China) with 95% purity. All the peptides were acetylated at the N-terminus and amidated at the C-terminus to mimic the native peptide structure. The peptides were dissolved in PBS to prepare 1 mM stock solutions for *in vitro* experiments and 5 mg/ml stock solutions for *in vivo* experiments.

### Protein expression and purification

The ddRFP biosensor (ddRFP) protein was obtained as previously described [7], *E.* coli BL21 (DE3) cells harboring His6-pET28a-ddRFP biosensor plasmid were grown in LB medium with 50 µg/mL Kanamycin. When optical density at 600 nm (OD600) reached at 1.0, the proteins were induced overnight at 16°C with 0.01 mM isopropyl β-D-1-thiogalactopyranoside (IPTG, #18070). The bacteria were lysed in buffer containing 0.05 M Tris-HCl (pH 8.0), 0.3 M NaCl. The proteins were then purified by Ni-NTA beads followed by washing with the buffer containing 0.02 M Tris-HCl (pH 7.5), 0.5 M NaCl and 0.025 M imidazole and eluting with the buffer containing 0.02 M Tris-HCl (pH 7.5), 0.15 M NaCl and 250mM imidazole. Then, the proteins were further purified by Hiload 16/600 Superdex 200pg gel-filtration chromatography with running buffer containing 20 mM Tris-HCl (pH 7.5), 150 mM NaCl. The purified proteins were concentrated to ~20 mg/mL and frozen in liquid nitrogen immediately. All purified proteins were stored in running buffer with 5 mM dithiothreitol (DTT).

### ddRFP biosensor specificity assay

Sixty microliter mixtures containing 10 μM 3C protease (30 μL/well) and ddRFP biosensor dilutions (30 μL/well) were dispensed into a black 96-well microplate. The RFU value was monitored via a microplate reader (λex = 535 nm; λem = 605 nm). The enzyme kinetics parameters were calculated via the Michaelis-Menten equation via GraphPad Prism (version 9.0).

### Peptide inhibition cleavage assay

For recombinant proteins *in vitro*, 20 μg pGSDMA^FL^, pcGAS^FL^, or sPro-IL-1β^FL^ recombinant protein incubated with 10 μg SVV 3C and different amounts of peptide (0.01, 0.1, 0.5, 1, 5, 10 μM) in a 25-μL reaction containing 50 mM HEPES (PH 7.5), 3 mM EDTA, 150 mM NaCl, 0.005% (vol/vol) Tween-20 and 10 mM DTT at 37°C for 2 h. The cleavage reaction was terminated by adding SDS-PAGE loading dye, followed analyzed by SDS-PAGE.

For cells analysis, HEK-293T cells were seeded into 6-well plates. When the cells were pre-treated with different dose of peptide for 6 h, a plasmid encoding V5-pGSDMA, Myc-pcGAS or sPro-IL-1β-HA and a plasmid encoding FLAG-SVV 3C were co-transfected for 24 h with Lipo2000 (Invitrogen, #11668019). When the plasmids were co-transfected into cells for 24 h, followed by treatment with different amounts of peptide for 6 h. Cells were then lysed with NP-40 (Solarbio, #N8032) containing protease inhibitors (Solarbio, # P6730) and analyzed by Western blot.

### FITC-peptide permeation assay

iPAMs and PK-15 cells were seeded into 24-well plates containing slides. When confluence reached up to 60–70%, the cells were treated with the FITC-peptide. For fluorescence analysis, cells were washed with PBS and fixed for 30 min in 4% paraformaldehyde (Solarbio, #P1110) and images were collected and analyzed by NIS-Elements AR. For flow cytometry analysis, cells were washed and analyzed GFP positive cells on an LSR Fortessa flow cytometer (BD Biosciences, USA) using FlowJo software (BD Biosciences, USA).

### Peptide stability assay

For the pH stability assay, P5 were pre-incubated in buffers of different pH values (3, 6.5, 7, 7.5, or 10). The treated peptide was then subjected to an *in vitro* cleavage assay in a 25-μL reaction containing 20 μg ddRFP recombinant protein, 10 μg SVV 3C, and 40 μM peptide in reaction buffer consisting of 50 mM HEPES (PH 7.5), 3 mM EDTA, 150 mM NaCl, 0.005% (vol/vol) Tween-20 and 10 mM DTT. Reactions wer incubated at 37°C for 2 h and terminated by addition of SDS-PAGE loading dye, followed by SDS-PAGE analysis. For heat stability assay, P5 was pre-incubated at different temperatures (25, 37, 40, or 60°C) for 2 h. The pretreated peptide was then added to a 25-μL cleavage reaction containing 20 μg ddRFP recombinant protein, 10 μg SVV 3C, and 40 μM peptide in the reaction buffer described above. Reactions were incubated at 37°C for 2 h and analyzed by SDS–PAGE as described above. For the serum stability assay, P5 were incubated with 50% porcine serum at 37°C for 0, 2, 4, 8, or12 h. After incubation, an equal volume of 7 M urea was added, and samples were heated at 95°C for 2 min to terminate serum-mediated degradation. The treated peptide was then used in a 25-μL cleavage reaction containing 20 μg recombinant ddRFP protein, 10 μg SVV 3C protease, and 40 μM peptide, followed by incubation at 37°C for 2 h. Reactions were terminated by addition of 10% trichloroacetic acid (TCA) and analyzed by SDS–PAGE.

### Enzyme-linked immunosorbent assay (ELISA)

PAMs were seeded into 6-well plates and were pre-treated with peptide for 6 h, then infected with SVV (MOI = 0.1) for 18 h, followed stimulated with poly (dA:dT) for 12 h. The secreted 2′3′-cGAMP (501700, Cayman) and IFN-α (819120921, Thermo Scientific) in cell culture medium from peptide-treated cells were analyzed with ELISA kits following the manufacturer’s instruction.

### IL-1β detection

To measure IL-1β release, cell culture supernatants were collected and analyzed using Porcine IL-1β ELISA KIT (Solarbio, #SEKP-0001).

### Cell viability assay

iPAMs were seeded in a 96-well plate, then treated with the indicated dose of peptides for 6 h. Static bright-field cell images were analyzed using Falcon S400, intelligent cell imaging, and analysis system (Alicelligent Technologies). Meanwhile, MTT assay was used to detect the cell viabilities. Cells were added 10 μL MTT solution (Solarbio, #M1025) to per 96-well plates and incubated in a cell incubator for 4 h. Absorbance at 490 nm was recorded using a microplate reader (Bio-Rad, USA). Relative viability was calculated as the ratio of the average absorbance of each treatment group to the control group after correction by the null group.

### Confocal microscopy

HeLa cells were transfected with SVV 3C for 18 h and then treated with FITC-labeled P5 for 6 h. Cells were fixed with 4% paraformaldehyde at room temperature for 15 min and subsequently permeabilized with 0.1% Triton X-100. To assess the co-localization of P5 with SVV 3C, cells were stained with an anti-FLAG antibody. Confocal microscopy (Nikon A1, Japan) was utilized for image acquisition and analysis.

### SVV replication assay

iPAMs were pre-treated with peptide for 6 h, then infected with SVV (MOI = 0.1) for different time points to determine the mRNA and protein levels of SVV.

For RT-PCR, RNA was extracted from whole-cell lysates with RNA simple Total RNA Kit (TIANGEN, #DP419) and reversely transcribed to cDNA with HiScript II Q RT SuperMix for qPCR (+gDNA wiper) Kit (Vazyme, #R223-01). The qPCR was performed using ChamQ SYBR qPCR Master Mix (Vazyme, #Q712-02).

For western blotting, cells were lysed with IP lysis buffer (Beyotime, #P0013) for 30 min on ice. Whole-cell lysates or immunoprecipitated extracts were then separated by 10% SDS-PAGE gels (EpiZyme, #PG112) and transferred onto PVDF membrane (Millipore) for immunoblotting with specific antibodies.

For flow cytometry analysis, cells were washed and analyzed GFP positive cells on an LSR Fortessa flow cytometer (BD Biosciences, USA) using FlowJo software (BD Biosciences, USA).

### Liquid-liquid phase separation (LLPS) assay in *vitro*

The purified recombinant EGFP-pcGAS protein was mixed at indicated concentration with LLPS buffer (1 mg/mL BSA, 20 mM Tris-HCl, 150 mM NaCl and 10% Ficoll), followed by incubating with indicated dsDNA, SVV 3C protein and peptide for 5 min at 37°C. The mixture was pipetted onto a glass bottom dish and images were collected and analyzed by NIS-Elements AR.

### Reverse transcription and quantitative real-time PCR (RT-qPCR)

Total RNA was extracted by a simple total RNA kit (TIANGEN, #DP419) and was reversely transcribed to cDNA using HiScript II Q RT SuperMix (Vazyme, #R223-01) according to the manufacturer’s protocol. qPCR was performed in triplicated determinants with 2 × Taq Pro Universal SYBR qPCR Master Mix (Vazyme, #Q712-02) on a Light Cycler 480 II system (Roche, Switzerland). Relative gene expression levels were determined based on the cycle threshold (ΔΔCT) method and normalized to glyceraldehyde-3-phosphate dehydrogenase (GAPDH) expression. The sequences of qPCR primer sequences were as follows: sus GAPDH-F: ACATGGCCTCCAAGGAGTAAGA, sus GAPDH-R: GACGCCTGCTTCACCACCTTCT; sus *Ifnb*-F: TGCATCCTCCAAATCGCTCT, sus *Ifnb*-R: ATTGAGGAGTCCCAGGCAAC; sus *Isg54*-F: GCACAGCAATCATGAGTGAGAC, sus *Isg54*-R: CTGGCCCCTGCAGTCTTTTA; sus *Isg15*-F: GCCTTCCAGCAGCGTCT, sus *Isg15*-R: GCGTTGCTGCGACCCT; SVV-*VP1*-F: AACCGGCTGTGTTTGCTAGAG, SVV-*VP1*-R: GAACTCGCAGACCACACCAA; SVV-*3C*-F: GAGCCTTTCCAGACGGTTCA, SVV-*3C*-R: CGTAACTAGCCGAAACGCCA.

### The MicroScale thermophoresis assay (MST)

SVV 3C WT or H48A proteins were serially diluted with deionized water. Then, FITC-P5, diluted with a buffer (1 mg/mL BSA, 0.05% Tween 20 in PBS), was incubated with SVV 3C WT or H48A proteins at room temperature in a total volume of 20 μL. The mixture was loaded into Monolith NT.115 capillaries. Binding affinity analysis was performed using a NanoTemper Monolith NT.115 instrument, and Kd values were obtained using MO. Affinity Analysis software.

### Sequence alignments

We collected amino acid sequences of pGSDMA (GenBank: XM_003131497.3) and other GSDMA homologs from hGSDMA (GenBank: XM_006721832.4), mouse GSDMA_1 (GenBank: XM_006533850.1), and rhesus monkey GSDMA (GenBank: XM_015119551.2). Clustal OMEGAonline software (https://www.ebi.ac.uk/Tools/msa/clustalo/) was used to perform the multiple-sequence alignment.

### Computational modeling

The all-atom MD simulations of SVV 3C (PDB: 6L0T) and P5 peptide were performed using HADDOCK2.4 on the website (https://wenmr.science.uu.nl/haddock2.4/).

### Statistical data analysis

All the graphs and relevant statistical tests used in the work were created by GraphPad software (v9.0). Data were expressed as mean ± SD and statistically analyzed with a two-tailed unpaired Student’s t-test. The *p* values of <0.05 were considered significant. *p < 0.05, **p < 0.01, ***p < 0.001, ****p < 0.0001. ns, no significance.

## Supporting information

S1 Fig Purification of ddRFP biosensor protein. (A) Alignment of porcine GSDMA (pGSDMA, Genbank: XM_003131497.3), human GSDMA (hGADMA, Genbank: XM_006721832.4), mouse GSDMA_1 (Genbank: XM_006533850.1) and rhesus monkey GSDMA (Genbank: XM_015119551.2) using Clustal Omega algorithm. (B and C) The detection of refolding of ddRFP biosensor protein by Ni-NTA (B) and gel-filtration chromatography (C).
(TIF)

S2 Fig Cleavage sites of SVV 3C in pGSDMA, pcGAS and sPro-IL-1β. (A) The Q187 and G188 were displayed in the modeling structure of pGSDMA using PyMOL software. (B) The W137 and Q140 amino acids were displayed in the structure of pcGAS (PDB: 4JLX) using PyMOL analysis software. (C) The L124 and Q125 were displayed in the modeling structure of sPro-IL-1β using PyMOL software.
(TIF)

S3 Fig Screening peptide inhibitors targeting 3C protease. SDS-PAGE analysis of 3C protease inhibition by engineered peptides. Reaction mixtures containing 20 μg ddRFP substrate, 10 μg 3C protease, and peptides (0.1 or 10 μM) were incubated at 37°C for 2 h.
(TIF)

S4 Fig Membrane permeability and cytotoxicity profiling of P5 peptide. (A) iPAMs were treated with the FITC-peptide (0.1-20 μM) for 6 h. Flow cytometry analysis the number of FITC-positive cells. Quantification of FITC⁺ cell percentage in right. (B) iPAMs were treated with the FITC-peptide (10 μM) for 1, 2, 4, 6 and 8 h. Flow cytometry analysis the number of FITC-positive cells. Time-course quantification of FITC⁺ cells in right. (C) PK-15 cells were treated with the FITC-peptide (0.1-20 μM) for 10 h. The number of FITC-positive cells was analyzed by fluorescence microscopy. Scale bar, 50 µm. (D) PK-15 cells were treated with the FITC-peptide (10 μM) for 1, 2, 4, 6 and 8 h. The number of FITC-positive cells was analyzed by fluorescence microscopy. Scale bar, 50 µm. (E) iPAMs were treated with increase doses of P5 peptide for 10 h. Morphological change characteristics of cells were visualized using light microscopy. Scale bar, 50 µm.
(TIF)

S5 Fig The specific inhibitory activity of P5 against 3C protease cleavage *in vitro.* (A) Schematic of the P5 peptide inhibition assay in *vitro*. Created in BioRender. Yin, H. (2026) https://BioRender.com/2jc9zb5. (B, C and D) The samples containing 20 μg pGSDMA^FL^, pcGAS^FL^ and sPro-IL-1β^FL^ were mixed with the 10 μg 3C and P5 peptide (0.1, 1, 5, 10, 40 μM) for 2 h at 37°C. SDS-PAGE analysis the P5 effects on pGSDMA (B), pcGAS (C) and sPro-IL-1β (D) cleavage.
(TIF)

S6 Fig Reconstitution of porcine cGAS-DNA phase separation *in vitro.* (A) Phase separation was induced with EGFP-pcGAS (10 μM) and 45 bp dsDNA (5 μM) in buffer with varying Ficoll concentration. Scale bar, 10 μm. (B and C) Fluorescence recovery after photobleaching (FRAP) of pcGAS-DNA phase separation condensates. Bleaching was performed at the indicated time points after pcGAS (10 μM) and DNA (5 μM) were mixed and the recovery was allowed to occur at 25°C. Scale bar, 1 μm. The maximal fluorescence intensity was normalized to 1. Quantitative analysis of recovery kinetics is shown in C.
(TIF)

S7 Fig P5 inhibits SVV 3C protease-mediated cleavage of EGFP-pcGAS. (A) The samples containing 10 μM EGFP-pcGAS were mixed with the 5 μM Cy3-dsDNA, 10 μM SVV 3C and 10 μM P5 for 6 min. Cleavage of EGFP-pcGAS was analyzed by western blot. (B) Samples containing 10 μM EGFP-cGAS, 5 μM Cy3-dsDNA, 10 μM 3C protease, and P5 (0.1, 1, or 10 μM) were incubated for 12 min. Cleavage of EGFP-pcGAS was analyzed by western blot.
(TIF)

S8 Fig SVV 3C suppresses cGAS-mediated IFN-I production. (A, B and C) iPAMs were transfected with increasing doses of SVV 3C for 24 h. Cells were stimulated with poly (dA:dT) (1 μg/mL). After another 12 h, cells were harvested for RNA extraction and RT-PCR analysis of *Ifnb* (A)*, Isg15* (B) *and Isg54* (C) expression. Data are represented as means ± SD from three biological replicates. *p < 0.05, **p < 0.01, ***p < 0.001, Student’s *t*-test.
(TIF)

S9 Fig P5 restores dsDNA-Induced IFN-I production by suppressing SVV 3C protease activity. (A, B and C) iPAMs were treated with increasing doses of P5 for 6 h, and then transfected with SVV 3C. After 12 h, cells were stimulated with poly (dA:dT) at a concentration of 1 μg/mL for 12 h. Cells were harvested for RNA extraction and RT-PCR analysis of *Ifnb* (A) and downstream *Isg15* (B) and *Isg54* (C) mRNA expression. (D, E and F) PK-15 cells were treated as above. Cells were harvested for RNA extraction and RT-PCR analysis of *Ifnb* (D) and downstream *Isg15* (E) and *Isg54* (F) mRNA expression. Data are represented as means ± SD from three biological replicates. ns, no significance, **p < 0.01, ***p < 0.001, ****p < 0.0001, Student’s *t*-test.
(TIF)

S10 Fig P5 restores dsRNA-Induced IFN-I production by suppressing SVV 3C protease activity. (A, B and C) iPAMs were treated with increasing concentrations of P5 for 6 h and then transfected with SVV 3C. After 12 h, cells were stimulated with poly (I:C) at a concentration of 1 μg/mL for 12 h. Cells were then harvested for RNA extraction and quantitative RT-PCR analysis of *Ifnb* (A) and downstream *Isg15* (B) and *Isg54* (C) mRNA expression. Data were represented as means ± SD from three biological replicates. ns, no significance, *p < 0.05, **p < 0.01, ***p < 0.001, ****p < 0.0001, Student’s *t*-test.
(TIF)
